# The role of rhizosphere phages in soil health

**DOI:** 10.1093/femsec/fiae052

**Published:** 2024-04-27

**Authors:** Xiaofang Wang, Yike Tang, Xiufeng Yue, Shuo Wang, Keming Yang, Yangchun Xu, Qirong Shen, Ville-Petri Friman, Zhong Wei

**Affiliations:** Jiangsu provincial key lab for solid organic waste utilization, Key lab of organic-based fertilizers of China，Jiangsu Collaborative Innovation Center for Solid Organic Wastes, Educational Ministry Engineering Center of Resource-saving fertilizers, Nanjing Agricultural University, Nanjing 210095, China; Jiangsu provincial key lab for solid organic waste utilization, Key lab of organic-based fertilizers of China，Jiangsu Collaborative Innovation Center for Solid Organic Wastes, Educational Ministry Engineering Center of Resource-saving fertilizers, Nanjing Agricultural University, Nanjing 210095, China; Jiangsu provincial key lab for solid organic waste utilization, Key lab of organic-based fertilizers of China，Jiangsu Collaborative Innovation Center for Solid Organic Wastes, Educational Ministry Engineering Center of Resource-saving fertilizers, Nanjing Agricultural University, Nanjing 210095, China; Jiangsu provincial key lab for solid organic waste utilization, Key lab of organic-based fertilizers of China，Jiangsu Collaborative Innovation Center for Solid Organic Wastes, Educational Ministry Engineering Center of Resource-saving fertilizers, Nanjing Agricultural University, Nanjing 210095, China; Jiangsu provincial key lab for solid organic waste utilization, Key lab of organic-based fertilizers of China，Jiangsu Collaborative Innovation Center for Solid Organic Wastes, Educational Ministry Engineering Center of Resource-saving fertilizers, Nanjing Agricultural University, Nanjing 210095, China; Jiangsu provincial key lab for solid organic waste utilization, Key lab of organic-based fertilizers of China，Jiangsu Collaborative Innovation Center for Solid Organic Wastes, Educational Ministry Engineering Center of Resource-saving fertilizers, Nanjing Agricultural University, Nanjing 210095, China; Jiangsu provincial key lab for solid organic waste utilization, Key lab of organic-based fertilizers of China，Jiangsu Collaborative Innovation Center for Solid Organic Wastes, Educational Ministry Engineering Center of Resource-saving fertilizers, Nanjing Agricultural University, Nanjing 210095, China; Jiangsu provincial key lab for solid organic waste utilization, Key lab of organic-based fertilizers of China，Jiangsu Collaborative Innovation Center for Solid Organic Wastes, Educational Ministry Engineering Center of Resource-saving fertilizers, Nanjing Agricultural University, Nanjing 210095, China; Department of Microbiology, University of Helsinki, 00014 Helsinki, Finland; Jiangsu provincial key lab for solid organic waste utilization, Key lab of organic-based fertilizers of China，Jiangsu Collaborative Innovation Center for Solid Organic Wastes, Educational Ministry Engineering Center of Resource-saving fertilizers, Nanjing Agricultural University, Nanjing 210095, China

**Keywords:** coevolution, microbial ecology, microbiome diversity, resistance, rhizosphere phages, soil health

## Abstract

While the One Health framework has emphasized the importance of soil microbiomes for plant and human health, one of the most diverse and abundant groups—bacterial viruses, i.e. phages—has been mostly neglected. This perspective reviews the significance of phages for plant health in rhizosphere and explores their ecological and evolutionary impacts on soil ecosystems. We first summarize our current understanding of the diversity and ecological roles of phages in soil microbiomes in terms of nutrient cycling, top-down density regulation, and pathogen suppression. We then consider how phages drive bacterial evolution in soils by promoting horizontal gene transfer, encoding auxiliary metabolic genes that increase host bacterial fitness, and selecting for phage-resistant mutants with altered ecology due to trade-offs with pathogen competitiveness and virulence. Finally, we consider challenges and avenues for phage research in soil ecosystems and how to elucidate the significance of phages for microbial ecology and evolution and soil ecosystem functioning in the future. We conclude that similar to bacteria, phages likely play important roles in connecting different One Health compartments, affecting microbiome diversity and functions in soils. From the applied perspective, phages could offer novel approaches to modulate and optimize microbial and microbe–plant interactions to enhance soil health.

## Teaser

We outline the significance of soil phages for plant health within the rhizosphere, delving into their ecological and evolutionary impacts on soil biodiversity and ecosystem functioning.

## Introduction

The One Health concept underscores the interconnectedness of human, animal, plant, and environmental health, emphasizing their interdependence (Banerjee and van der Heijden 2022). Originally, the One Health concept centered on the transmission of zoonotic pathogens, vectors of pathogens, and movement and persistence of antibiotic resistance genes (ARGs) across environments (Destoumieux-Garzón et al. 2018). Recently, soils have been proposed to be a critical compartment linking human, animal, and plant health via the sharing of microorganisms (Banerjee and van der Heijden 2022). Further, soil microbiomes have been estimated to provide over 40 functions linked with plant growth, nutrient uptake and cycling, provision of essential ecosystem services, and suppression of pathogens, highlighting their importance in One Health framework (Lehmann et al. 2020, Banerjee and van der Heijden 2022). Especially, plant rhizosphere is considered to play an important role in maintaining high microbial diversity, which has been positively associated with soil health, crop yields, and ecosystem functioning (Fierer 2017, Saleem et al. 2019, Wagg et al. 2019, Banerjee and van der Heijden 2022). The benefits of soil biodiversity can be mediated via mutualistic interactions between plants and microorganisms, resulting in improved plant growth (Trivedi et al. 2020, Li et al. 2021a). Additionally, high microbial diversity can be beneficial in preventing pathogen invasions and dominance, reducing economic losses on agricultural production (Strange and Scott 2005, Delgado-Baquerizo et al. 2020, Zheng et al. 2020). Such pathogen suppression can be driven by several different groups of microorganisms, including bacteria, fungi, protists, and bacteria-specific viruses—phages (Buee et al. 2009, Wei et al. 2015, Wang et al. 2019, Xiong et al. 2020). Of these microbes, phages are the most understudied group even though they are estimated to be the most abundant entities on Earth (estimate 10^31^) (Breitbart and Rohwer 2005, Srinivasiah et al. 2013), constituting a significant portion of virus-like particles (VLPs) in the environment (Breitbart and Rohwer 2005). Previous research has demonstrated that phages play crucial roles in influencing microbial abundances, diversity, and functioning of ecosystems across aquatic, human, and terrestrial environments (Wilhelm and Suttle 1999, Brum et al. 2016, Argov et al. 2017). However, the role of phages in soil, rhizosphere and plant health remains relatively understudied. In this perspective, we review the significance of phages for soil health and microbial diversity within the One Health framework by focusing on both their ecological and evolutionary roles in rhizosphere microbiomes.

### The diversity of phages in the plant rhizosphere

While the significance of phages for microbial ecology and evolution has been extensively studied especially in marine ecosystems (Diaz-Munoz and Koskella 2014, Breitbart et al. 2018), the knowledge of phage diversity and their roles in soil and plant rhizosphere microbiomes is still relatively limited. In contrast to aquatic environments, soils exhibit higher heterogeneity due to variations in soil particle size, nutrient composition, soil biota, and plant community diversity and composition (Sharma et al. 2011, 2016). Soils exhibit higher heterogeneity compared to aquatic environments, consisting of solid, liquid, and gaseous phases, which can considerably vary across space and time (Moldrup et al. 2001). Specifically, soil particle and aggregate sizes, and pore spaces affect hydraulic connectivity (Tecon and Or 2017, Roux and Emerson 2022), which influences the movement of phages and bacteria within the soil matrix along with water (Mckay et al. 1993, Marsh and Wellington 1994, Philippot et al. 2023). Well-aggregated soils typically possess larger pores and better water infiltration compared to poorly structured ones, potentially facilitating greater dispersal and distribution of phages. Similarly, soils with larger particles (e.g. sandy soils) might allow easier phage movement owing to greater pore space and better water flow. In support of this, previous studies have shown that viral communities respond rapidly to wetting events, resulting in a significant increase in viral richness (Santos-Medellin et al. 2023). Additionally, the adsorption of viruses to soil particles, such as colloidal surfaces, has been found to enhance viral persistence and support higher viral abundances (Lipson and Stotzky 1986, Zhuang and Jin 2003). It has also been shown that viral communities undergo shifts along thawing permafrost peatland soils, with correlations observed with host community composition, pH, soil moisture content, and soil depth (Emerson et al. 2018). As a result, the abundance and diversity of bacteria and phages varies spatially between and within terrestrial habitats and soil types (Williamson et al. 2017, Geisen et al. 2019), resulting in relatively higher phage diversity in soils compared to aquatic environments (Guemes et al. 2016). The estimated number of distinct viral genotypes (richness) identified in soils varies between 1000 and 1 000 000 depending on the soil type (Ashelford et al. 2003, Williamson et al. 2005, Reavy et al. 2015, Williamson et al. 2017). In contrast, viral richness estimates vary from 532 to 129 000 for marine, and between 400 and 40 000 viral types for freshwater systems (Green et al. 2015). Moreover, phage abundances have been estimated to be relatively more homogeneous in seawater (between 10^5^ and 10^7^ particles per millilitre) compared to soils (between 10^3^ and 10^9^ particles per gram of soil) (Graham et al. 2019). While these diversity and abundance estimates are highly variable, likely due to variation in abiotic and biotic factors between and within both habitats, they suggest that terrestrial environments harbour higher taxonomic viral diversity. Phages also exhibit extensive morphological diversity, including tailed, nontailed, and filamentous forms (Nobrega et al. 2018), show high variability in nucleic acids composition (dsDNA, ssDNA, dsRNA, and ssRNA) (Dion et al. 2020) and have adopted various life cycles, ranging from lytic to lysogenic and chronic (Simmonds et al. 2017, Ofir and Sorek 2018). Especially, temperate phages capable of either lysing the host cell or integrating and replicating as part of the host genome are prevalent in soils (Argov et al. 2017, Howard-Varona et al. 2017, Sharma et al. 2019). When integrated into the host genome during lysogeny, temperate phages can facilitate their host survival, thus sustaining phage and bacterial population densities in soils (Williamson et al. 2002, Weinbauer et al. 2003, Chibani-Chennoufi et al. 2004).

Soil phage diversity also varies spatially within a given location. One important factor shaping local phage community diversity is the presence of plants. For example, it has been shown that viral-to-bacterial ratios differ between the rhizosphere and bulk soils and that lower bacterial abundances in the bulk soil are thought to be the contributing factor explaining relatively lower phage diversity (Swanson et al. 2009). Similarly, Bi et al. (2021) identified significant differences in phage community composition between rhizosphere and bulk soils, which were mainly attributed to the absence of plant root exudates and plant litter in the bulk soil that acts as nutrient sources for bacteria (Haichar et al. 2008, Zhalnina et al. 2018), promoting also higher phage abundances and diversity. Moreover, rhizosphere phage communities vary between different plant species, due to differences in plant root exudate composition (Zhalnina et al. 2018), which can determine the composition and abundances of plant-associated bacterial microbiomes (Wang et al. 2022). In addition to spatial variation, the diversity and activity of phages can show dynamic temporal changes, driven by seasonal fluctuations in temperature (Coclet et al. 2023), soil moisture (Santos-Medellin et al. 2023), plant growth and development (Yang et al. 2023), and anthropogenic factors such as crop management (Muscatt et al. 2022). These external factors can also influence the intrinsic dynamics between phages and their host, e.g. by triggering population fluctuations and ecological succession in microbial communities, which can feed back to ecosystem functioning (Liao et al. 2023, Santos-Medellin et al. 2023). The emergence of novel methodologies for purifying, extracting, and quantifying phage assemblages from soils (Williamson et al. 2003), coupled with advancements in metagenomic sequencing, has paved the way for a more in-depth exploration of the true soil phage diversity (Paez-Espino et al. 2019, Koonin and Yutin 2020, Roux and Emerson 2022, Mabrouk et al. 2023), while metatranscriptomic approaches have opened the door to study phage activity and RNA phage diversity (Callanan et al. 2020, Neri et al. 2022). With these methods, we are continuously discovering novel phage diversity that does not exist in the current databases (Roux and Emerson 2022), increasing our understanding on the significance and various roles phages play for soil and rhizosphere microbiome diversity and functioning in terrestrial ecosystems.

### Ecological impacts of rhizosphere phages in soil ecosystems

#### The viral shunt: the role of phages in soil nutrient cycle

**Figure 1.**
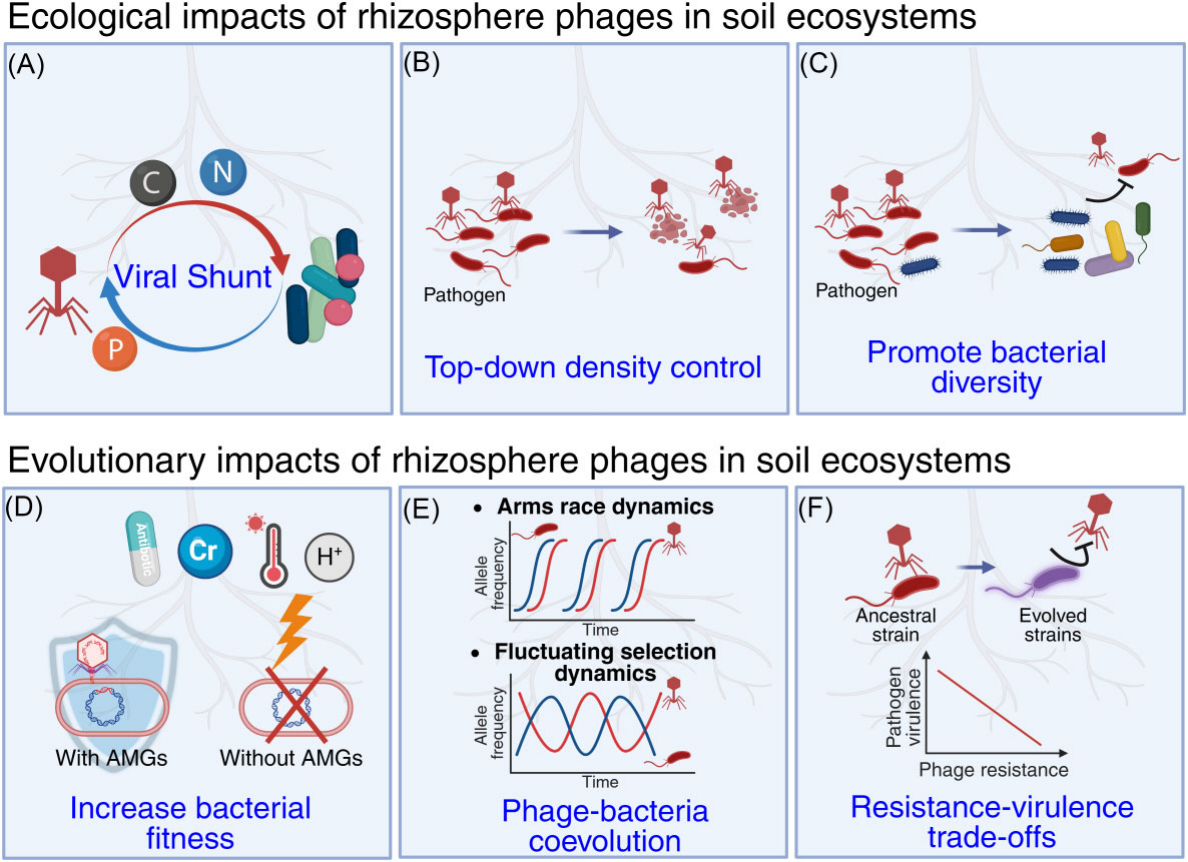
Summary of ecological and evolutionary roles of rhizosphere phages in soil health. The ecological impacts of rhizosphere phages on soil ecosystems include phage effects on nutrient cycling via lysis of bacterial cells (A), top-down density regulation of bacteria (B) and positive phage effects on microbial community diversity, stability, and composition via Kill-the-Winner dynamics (C). The evolutionary impacts of rhizosphere phages on soil ecosystems include horizontal gene transfer, including phage-encoded AMG that increase host bacterial fitness (D), phage–bacteria coevolution, and (E) selection for phage-resistant mutants with altered ecology due to genetic correlations such as trade-offs with pathogen virulence *in planta* (F).

Phages play a crucial role in impacting the mortality of bacteria by lysing and releasing host cell contents into the environment, with significant consequences for the global cycling of nutrients, energy flow, and food web dynamics—a phenomenon known as the “viral shunt” in aquatic systems (Brum and Sullivan 2015, Breitbart et al. 2018) (Fig. 1A). For instance, marine phages are estimated to lyse approximately one-third of ocean microorganisms per day, liberating carbon at a globally significant scale (Suttle 2005, 2007, Guidi et al. 2016). In contrast, there are still significant knowledge gaps regarding how soil phages contribute to food webs, decomposition of organic matter, carbon and nutrient cycling, greenhouse gas emissions, and agricultural productivity. Virulent phages can control host population abundances, thereby influencing rates of microbially mediated processes linked with the cycling of carbon, nitrogen, sulfur, and phosphorus in soils (Williamson et al. 2005, Kimura et al. 2008, Allen et al. 2010, Helsley et al. 2014). For instance, phages that infect nitrogen-fixing rhizobia bacteria, have been shown to reduce nodulation by phage-sensitive rhizobia, thus influencing nitrogen fixation in legume–rhizobium symbiosis (Evans et al. 1979, Sharma et al. 2002). Recently, temporal changes in phage communities were linked with nutrient cycling during composting, where phages specific to mesophilic and thermophilic bacteria tracked their host densities, triggering bacteria-phage community succession via top-down control (Liao et al. 2023). Crucially, nutrient turnover correlated positively with virus–host ratio, indicative of a positive relationship between ecosystem functioning, viral abundances, and viral activity (Liao et al. 2023). Moreover, viruses specific to mesophilic bacteria encoded and expressed several auxiliary metabolic genes (AMGs) linked to carbon cycling, impacting nutrient turnover alongside bacteria (Liao et al. 2023). Both temperate phages and virulent phages can encode AMGs, which can affect the host cell metabolism in ways that promote viral replication and host survival (Howard-Varona et al. 2020, Puxty and Millard 2023). Phages are hence likely to influence nutrient cycling by metabolically reprogramming their host bacteria through the expression of virus-carried AMGs (Breitbart 2012) linked with carbon metabolism and degradation of soil organic matter (Trubl et al. 2018, Wu et al. 2021). It remains yet unclear how phage-mediated nutrient cycling in the soil cascades through microbial food webs, affecting the plant growth and fertility of soils. Both lab- and field-scale experiments, are hence required to directly test how viruses might shape the soil microbiome composition and plant growth by turning over bacterial biomass via lysis and by encoding AMGs associated with nutrient cycling.

#### Virulent phages: suppression of soil-borne bacterial pathogens via top-down density control

Virulent phages obligately infect and lyse their host cells, exerting significant effects on host cell densities and triggering competitive dynamics within microbial communities (Morella et al. 2018) (Fig. 1B). As a result, virulent phages have been extensively explored for their potential as biological control agents, demonstrating successful applications in the control of plant and zoonotic pathogens in soils (Jones et al. 2007, Buttimer et al. 2017). Phages are also more likely to survive in the soil as long as their host bacteria are present compared to biocontrol bacteria that might have poor survival in the rhizosphere due to lack of vacant niche space and competition with native microbiota (Jones et al. 2007, Brodeur 2012, Meyer 2013, Buttimer et al. 2017, Kaminsky et al. 2019, AL-Ishaq et al. 2021). The earliest evidence of phage therapy applied to plant disease dates back to 1924 when the filtrate from rotten cabbage was used to inhibit the growth of *Xanthomonas campestris* (Mallmann and Hemstreet 1924). Since then, phage therapies have been used in the treatment of various phytopathogens, including *Pseudomonas syringae* (Frampton et al. 2014, Rombouts et al. 2016), *Ralstonia solanacearum* (Fujiwara et al. 2011, Wang et al. 2019), *Xanthomonas* spp. (Balogh et al. 2008), *Erwinia amylovora* (Schnabel and Jones 2001, Kim et al. 2020), as well as zoonotic pathogens present in soils, including *Salmonella anatum* (Gessel et al. 2004) and *Rhodococcus equi* (Salifu et al. 2013). Most of these studies predominantly examined the direct effects of virulent phages on the population densities of target pathogens through cell lysis. As a result, there is a growing interest in utilizing phage-derived proteins, such as endolysins, for pathogen biocontrol (O’Flaherty et al. 2009). Researchers have identified several techniques to enhance the efficacy of phage biocontrol, including optimizing the timing, frequency, and dosage of application (Iriarte et al. 2007, Cui et al. 2019, Li et al. 2020), employing phages as multiphage cocktails (Alvarez et al. 2019, Wang et al. 2019, Rabiey et al. 2020, Thapa Magar et al. 2022) or rationally combining phages with antibiotics (Torres-Barcelo and Hochberg 2016, Torres-Barcelo et al. 2016) or probiotics (Wang et al. 2017). Several products have been developed and already made commercially available, including AgriPhage^™^ and Erwiphage^™^ (Grace et al. 2021). However, most of these products target pathogens in the phyllosphere and no products targeting soil-borne pathogens are yet commercially available to our knowledge.

Beyond the ecological impacts of phage therapy in controlling pathogen densities, phages may also have indirect benefits for soil microbiome diversity by preventing pathogens from monopolizing the niche space, and potentially stabilizing interactions in rhizosphere microbiomes (Federici et al. 2020) (Fig. 1C). One previous study demonstrated that *R. solanacearum*-specific phage cocktail buffered the resident rhizosphere microbiota against changes induced by the pathogen invasion (Wang et al. 2019). In addition to affecting the bacterial community composition, the application of the phage cocktail also changed the potential functioning of the bacterial community by altering the proportion of taxa that exhibited facilitative or antagonistic pairwise interactions with the pathogen (Wang et al. 2019). These experimental findings are in line with other studies where healthy plants were associated with higher abundances of *R. solanacearum*-specific phages and a higher proportion of bacterial taxa exhibiting antagonism towards the pathogen (Wei et al. 2019, Yang et al. 2023). Interestingly, phages specific to bacterial taxa that showed antagonism towards *R. solanacearum*, had an indirect positive effect on plant disease by controlling the densities of antagonistic bacteria both in the lab and the greenhouse experiments (Yang et al. 2023). While phage effets might not always be so drastic on the surrounding microbiota (Magar et al. 2024), these findings suggest that phage efficacy could be context-dependent, shaping and being shaped by the resident microbiota present in the rhizosphere. Such complex ecological feedback and dynamics triggered by virulent phages are now only starting to be discovered and will have implications for microbial soil ecology beyond pathogen density control.

### Evolutionary impacts of rhizosphere phages in soil ecosystems

#### Temperate phages: horizontal transfer of AMGs in soils

Temperate phages are widely acknowledged for their role in mediating horizontal gene transfer between bacteria via transduction and phages could hence drive bacterial evolution by promoting recombination and provision of new genes (Lwoff 1953, Brussow et al. 2004, Bobay et al. 2014, De Paepe et al. 2014, Martin-Galiano and Garcia 2021). Earlier microcosm work has demonstrated high efficiency of phage transduction in soils (Zeph et al. 1988). Even though the rate of phage transduction in natural soils is still less well-understood, several examples exist on phage-mediated HGT and associated benefits for host bacteria in soils. For instance, phages in atrazine-contaminated soils were found to be able to acquire the *trzN* gene, encoding a chlorohydrolase required for atrazine catabolism (Ghosh et al. 2008). Additionally, Ross and Topp (2015) conducted transduction experiments using phages isolated from the soil to infect *Escherichia coli* K-12 and discovered that sublethal antibiotic concentrations could promote phage-mediated HGT of ARGs in agricultural soil microbiomes. These phage-encoded ARGs include multidrug resistance, polymyxin, and β-lactamase resistance genes (Lekunberri et al. 2017, Moon et al. 2020), that can help bacteria resist antimicrobials originating from anthropogenic or environmental sources when produced by plants, bacteria, or fungi. Phages carrying ARGs have been shown to be infective in propagation experiments, indicating their role as vehicles of ARG transmission between bacteria (Larranaga et al. 2018). While ARGs can be mobilized by phages (Torres-Barceló 2018), it is still, however, unclear how common this phenomenon is in soil microbiomes (Enault et al. 2017). Integration of temperate phages into bacterial genomes as prophages could also change bacterial gene expression, leading to loss of functioning of certain genes (Chen et al. 2020, Hsu et al. 2020, Zhou et al. 2023). For example, Davies et al. (2016) found that when transposable phage ɸ4 integrated randomly into the bacterial chromosome, it resulted in insertional inactivation of type IV pilus and Quorum Sensing-associated genes, which was adaptive. Lysogenic phages could further become ‘grounded’ if mutations in one or more phage genes result in the failure of prophage excision from the host genome, also referred to as ‘cryptic’ or defective prophages (Ramisetty and Sudhakari 2019). These defective prophages can further promote genome evolution through propensity for genetic variations including inversions, deletions, and insertions via horizontal gene transfer (Monteiro et al. 2019, Ramisetty and Sudhakari 2019). Moreover, temperate phages can enhance bacterial adaptability by increasing the mutation supply rate and thus help generating new genetic raw material for selection (Canchaya et al. 2004, Zhang et al. 2022).

In addition to facilitating horizontal gene transfer, temperate phages are also capable of altering host metabolism through the expression of AMGs (Yu et al. 2015, Sun et al. 2023a) (Fig. 1D). AMGs originate from bacterial cells but are carried by phages to enhance their own and their host’s fitness and can also contribute to the breadth of the phage host range (Sharon et al. 2011). In cyanophages, AMGs have been associated with functions such as photosynthesis, nucleic acid synthesis, metabolism, and stress tolerance (Thompson et al. 2011, Kelly et al. 2013, Enav et al. 2014). Soil environments are also important reservoirs for viruses that encode AMGs (Sun et al. 2023a). Phages can also provide their host bacteria beneficial traits through lysogenic conversion where they integrate into bacterial chromosome as prophages. These traits can, e.g. include virulence traits (Fortier and Sekulovic 2013, Matos et al. 2013, Taylor et al. 2019) or AMGs and different soil environments may enrich specific AMGs with diverse ecological functions. For example, phage AMGs have been associated with the breakdown of harmful pollutants, including atrazine degradation gene *trzN* in atrazine-contaminated soil (Ghosh et al. 2008), the arsenic resistance gene *arsC* in lysogenic soil viruses (Tang et al. 2019), and the virus-encoded L-2-haloacid dehalogenase gene (L-DEX) in organochlorine-contaminated soil (Zheng et al. 2022). Moreover, the presence of phage AMGs in vermicompost has been linked to both metabolism and pesticide biodegradation (Chao et al. 2023), while chromium-induced stress can enrich AMGs that contribute to microbial heavy metal detoxification and survival in stressful soil environments (Huang et al. 2021). Beyond helping host bacteria to survive in contaminated environments, phage AMGs can also be involved in the carbon and nitrogen cycling in agricultural soils (Roux and Emerson 2022), and participate in carbon and sulfur transformation in agricultural slurry (Cook et al. 2021). Some AMGs have also been associated with energy acquisition via oxidative respiration, degradation of organic matter, and plant-beneficial functions in the rhizosphere (Braga et al. 2021, Wu et al. 2021). Selection could, hence act on both host bacteria and prophages, enriching their frequencies if prophage improve host bacterial fitness relative to prophage-free cells.

Also, some plant-beneficial AMGs have been detected in phage genomes that possibly contributed to plant–microbe interactions (Braga et al. 2021). For example, succinoglycan and acetolactate biosynthesis play important roles in nodule formation and plant-growth promotion and have been found to be encoded by phages (Ryu et al. 2003, Mendis et al. 2016). Phage-encoded AMGs can also carry pathogenicity factors, such as effector proteins, that could help pathogenic bacteria to evade plant immunity (Greenrod et al. 2022). For example, it was recently shown that *hopAR1* effector protein is encoded by a prophage that can transmit these virulence factors between different *P. syringae* bacterial genotypes (Hulin et al. 2023). From the evolutionary perspective, selection could, thus act on both host bacteria and prophages, enriching their frequencies if prophages improve host bacterial fitness relative to prophage-free cells. A further investigation into the roles of phage AMGs in soil environments is needed to better understand their associated functions and evolutionary advantages for bacterial hosts and surrounding microbiota and plants.

#### Phage–bacteria coevolution in the rhizosphere

Rapid phage–bacteria coevolution plays a pivotal role in shaping the dynamics of microbial communities and ecosystem functioning in rhizosphere microbiomes (Koskella and Taylor 2018, Fields and Friman 2022). To resist phage infection and lysis, soil bacteria have developed a plethora of resistance and defence mechanisms, while phages have evolved numerous ways to overcome them, resulting in a long-term, coevolutionary arms race (Bernheim and Sorek 2020) (Fig. 1E). To initiate the infections, phages first need to adsorb to bacterial surfaces to inject their genetic material inside the bacterial cells. To escape this, bacteria have evolved numerous ways to prevent phage adsorption, including losing the phage receptor, reducing the expression of the receptor, modification of the receptor through mutations, or producing proteins that block phage adsorption by masking the receptor (Rostol and Marraffini 2019). Once the phage has adsorbed to the surface of the bacteria, bacteria can further alter the permeability of the surface, preventing the injection of phage DNA (Labrie et al. 2010). If phages are successful in infecting their DNA or RNA within bacterial cells, bacteria can activate a second line of defences to recognize and degrade the phage nucleic acids. In summary, these defence strategies can be divided into two steps: first detecting the infection of incoming phage with sensors, and second, the initiation of interference on phage reproduction cycle (Georjon and Bernheim 2023). These mechanisms include, e.g. restriction-modification (R-M) (Vasu and Nagaraja 2013) and the CRISPR–Cas systems (Barrangou et al. 2007) that recognize and destroy incoming phage nucleic acids. If phages can avoid the recognition by these systems and program the host to start synthesizing phage proteins, other bacterial defence systems, such as ToxIN, DarTG, and CBASS (Georjon and Bernheim 2023), can detect the viral proteins and trigger abortive cell death, which will eliminate infecting phages, promoting the survival of surrounding uninfected bacterial cells (Gao et al. 2022). For example, phage infections can activate toxin functions, leading to interference with bacterial DNA replication and protein translation, and prevention of phage reproduction. Alternatively, phage infection can trigger the production of cyclic oligonucleotide-based antiphage signaling system (CBASS), which activates effector protein that destroys the host cell and block phage propagation (Georjon and Bernheim 2023). However, this hierarchical activation is only as one potential explanation for the presence of multiple different types of defence systems, and alternatively or additionally, complementary and synergistic effect between defence systems, might enable bacteria to adopt flexible defence strategies against phages (Zhuang and Jin 2003).

Interestingly, antiphage defence systems can also be carried by prophages, which include repressor-mediated immunity, exclusion-like systems, and restriction mechanisms, that can offer protection against a broad range of related and unrelated viruses (Kita et al. 2003, Dedrick et al. 2017, Patel and Maxwell 2023). Owen et al. (2021) identified BstA protein—a family of prophage-encoded phage-defence proteins—in various Gram-negative bacteria, which enable prophages to defend host cells against exogenous phage attacks without sacrificing their ability to replicate lytically. Additionally, Patel et al. (2024) discovered that a prophage can encode Tab protein, which mediates the antiphage defence by blocking virion assembly of invading phages. Furthermore, prophages have been shown to serve as primary reservoirs and distributors of defence systems in *E. coli*, which can be located in specific genomic regions, i.e. defence system islands (Rousset et al. 2022, Vassallo et al. 2022).

Through coevolution, phages have evolved multiple counter adaptations to avoid bacterial defence systems or infect bacterial cells with mutated or alternative receptors. For example, phage λ that primarily uses the LamB as its receptor, has been shown to rapidly coevolve to bind more efficiently to this receptor and even evolve to recognize alternative *E. coli* receptor (OmpF) in response to *E. coli* resistance evolution (Meyer et al. 2012). Such phage coevolutionary changes are often achieved via changes in phage tail fibers and other host-recognition proteins (Nobrega et al. 2018, Altamirano and Barr 2021, Borin et al. 2023). When phage nucleic acids enter the host bacteria, phages can employ various antidefence system strategies, including antirestriction modification and anti-CRISPR proteins. For instance, in coliphage P1 (Myoviridae), the proteins DarA and DarB are coinjected into the host cell with the phage genome. Both proteins bind to phage DNA, masking type I R–M recognition sites, preventing the degradation of phage DNA (Atanasiu et al. 2002). Moreover, phages have evolved anti-CRISPR proteins (Acrs) that inhibit the cleavage of Cas proteins. For example, AcrIIC4 is a broad-spectrum Acr that binds between the two recognition domains of Cas9, REC1 and REC2, restricting the movement of the REC2 domain and thereby maintaining the integrity of its genome (Sun et al. 2023b). Previous studies have demonstrated that phage–bacteria coevolution follows fluctuating selection in the soil microcosms, where phages and bacteria adapt to their contemporary counterparts in time (Gomez and Buckling 2011). While a laboratory study showed that the rate of evolution increases with soil-inhabiting *Pseudomonas fluorescens* SBW25 in the presence of a phage (Pal et al. 2007), a follow-up microcosm study contradicted this finding, where the presence of virulent phages and a natural soil virome negatively affected the evolution of SBW25 (Gomez and Buckling 2013). While not specifically examining the coevolution between bacteria and phages, another study revealed that phages can rapidly select for resistant bacteria in the tomato rhizosphere, which leads to trade-off with bacterial growth and competitive ability (Wang et al. 2019). With the advancement of bioinformatic approaches, numerous novel defence systems and antidefence systems are continuously discovered (Gao et al. 2020, Nussenzweig and Marraffini 2020, Millman et al. 2022), and it is now becoming important to try to understand their significance, synergies and relative importance for phage–bacteria coevolution in natural environments, including rhizosphere. For example, as receptor and defence system-based resistances are not mutually exclusive (Alseth et al. 2019, Wang et al. 2023), it is important to quantify their costs and benefits in ecologically realistic contexts to assess their importance for phage–bacteria coevolution in terrestrial ecosystems. Understanding the underlying variation and rate of phage resistance evolution in agricultural environments is especially important for the long-term success of phage biocontrol applications.

#### The consequences of phage resistance evolution for bacterial fitness

Research thus far has demonstrated that bacteria can rapidly evolve resistance to phages in the soil (Gomez and Buckling 2011) and the rhizosphere (Wang et al. 2019). However, evolving resistance to phages often comes with costs due to loss or reduced functioning of associated receptor genes, which could change how bacteria interact with other microbes (Fig. 1F). For example, evolution of broad phage resistance of phytopathogenic *R. solanacearum* was shown to lead to relatively higher costs of resistance in terms of reduced pathogen growth and competitiveness with nonresistant, ancestral *R. solanacearum* genotype (Wang et al. 2019). Costs of resistance could also affect how bacteria interact with plants or behave in their environment because receptor mutations often occur in surface proteins that may also use to attach on plant surfaces or move and navigate in the rhizosphere or soil matrix (Addy et al. 2012, Ahmad et al. 2014, Narulita et al. 2016). For example, phage resistance mutations in genes encoding type IV pilus and type II secretion systems important for bacterial movement and secretion of exoenzymes, respectively, have been linked to both phage resistance and loss of virulence in *R. solanacearum* (Narulita et al. 2016, Xavier et al. 2022, Wang et al. 2023). Moreover, mutations in the quorum-sensing (QS) signalling receptor gene, *phcS*, have been shown to lead to phage resistance and loss of virulence in *R. solanacearum* even though the underlying molecular mechanisms remain unclear (Wang et al. 2023). Finally, also the upregulation of CBASS and type I restriction-modification phage defence systems in response to phage exposure were found to correlate with reduced expression of motility and virulence-associated genes, including pilus biosynthesis and type II and III secretion systems, in *R. solanacearum* (Wang et al. 2023). Together these findings suggest that both phage resistance mutations and upregulation of phage defence systems could result in trade-offs with pathogen virulence and fitness.

The evolution of phage resistance could also make bacteria more susceptible to other stresses. For example, phage-resistance mutations have been found to sensitize bacterial pathogens to antimicrobial compounds (Torres-Barcelo and Hochberg 2016), and in the clinical context, phage–antibiotic combinations have been found to have superior efficacy to mono-treatments because phage resistant bacteria became more sensitive to antibiotics due to mutations in genes that increase antibiotic efficacy (Chan et al. 2016, Altamirano et al. 2022). While the evolution of generalist resistance to both antibiotics and phages is also possible (Moulton‐Brown and Friman 2018, Burmeister and Turner 2020), similar sensitization of *R. solanacearum* to antibiotics produced by *Bacillus amyloliquefaciens* soil bacterium has been reported because of phage resistance evolution (Wang et al. 2017). If such trade-offs are more common among soil bacteria, phages could also indirectly affect antibiotics-mediated competition between different microbes in soil microbiomes. Finally, the magnitude of the cost of phage resistance is known to vary depending on the environmental conditions, such as nutrient availability, spatial structure, and the strength of resource competition (Brockhurst et al. 2003, Lopez-Pascua et al. 2010, Gomez and Buckling 2011, Gomez et al. 2015, Alseth et al. 2019, Chevallereau et al. 2022). As a result, the evolution of phage resistance could be constrained by its associated costs in complex rhizosphere microbiomes compared to more benign lab environments. More realistic experiments using soil and plant systems are, hence required to better understand the fitness costs of phage–bacteria coevolution for both partners in plant rhizosphere microbiomes.

### Challenges and avenues for future research

While research on phage genetics and molecular biology has been advancing in leaps and bounds along with the discovery of myriad of new defence systems (Georjon and Bernheim 2023), research on phage ecology and evolution in terrestrial environments is trailing back. While tracking interactions between focal species pairs, such as plant pathogenic bacteria and their specific phages (Gomez and Buckling 2011, Wang et al. 2019), has helped to understand pairwise coevolution in soils and the rhizosphere, this view is simplistic as most bacteria in the soils are likely to have their own specific phages, and hence potential to coevolve and interact with them. While metagenomic sequencing and separation of phage and bacteria fractions before sequencing will has helped to unravel the true phage diversity in terrestrial systems (Roux and Emerson 2022), it is still challenging to infer interactions based on sequence data (Wu et al. 2023). This will undoubtedly change when we discover more about the genetics and molecular biology underlying phage–bacteria interactions, the advancement of bioinformatics and computational techniques (Gaborieau et al. 2023) and the use of more realistic model ecosystems, such as rhizoboxes (Wei et al. 2019). The structure of soils also creates limits for understanding phage–bacteria interactions at different spatial scales. For example, it is difficult to determine phage–bacteria population and metapopulation borders. How far can phages migrate passively or by hitchhiking with their host bacteria? How long are phages able to persist in environments in the absence of their hosts? All these questions remain yet to be answered. Going forward, it is also important to employ omics techniques to understand the role of temperate phages for the horizontal transfer of AMGs and how temperate phages interact with other mobile genetic elements such as plasmids, conjugative elements, and phage satellites (Rocha and Bikard 2022). For example, what is the relative contribution of phages to the accessory genome of bacteria in terrestrial ecosystems? What are the key roles of phages for their host metabolism and how is phage diversity linked with microbial and terrestrial ecosystem diversity?

More work on how to potentially harness phage ecology and evolution for the benefit of phage applications and ecosystem functioning in terrestrial ecosystems is also needed. As phages are often specific to their target bacteria, they could potentially be used to precision-edit bacterial communities by removing specific bacterial taxa or functions (Wang et al. 2019). For example, in addition to targeting plant pathogenic bacteria, one could use phages to target bacteria that interact with the pathogen in the rhizosphere microbiomes (Li et al. 2021b, Yang et al. 2023) or target bacteria that carry ARGs that are located in conjugative plasmids, requiring pilus expression for their transmission, which makes them also susceptible to phage infections (Jalasvuori et al. 2011). Alternatively, it might be possible just to target specific key taxa known for ARG carriage or just reduce overall bacterial abundances using nonspecific phage communities as has been done with the treatment of sewage systems (Yu et al. 2017). In contrast to removing bacterial taxa or functions, temperate phages could also be used to introduce new beneficial functions in the soil microbiomes. For example, if plant growth-promoting AMGs are common among temperate phages, such phages might be used to deliver plant-beneficial functions into rhizosphere microbiomes to potentially improve plant health and crop yields. Such beneficial functions could be identified, e.g. from so-called suppressive soils that can constrain plant pathogen infections and promote plant growth (Garbeva et al. 2004, Peralta et al. 2018). Instead of identifying specific phage species, employing phage communities as part of rhizosphere soil transplants could be used as an initial screen to identify beneficial microbial communities. For example, soil transplant from healthy tomato plants was shown to constrain *R. solanacearum* pathogen invasion in the next tomato generation and this effect was likely driven by both pathogen-suppressing bacteria and *R. solanacearum*-specific phages present in the transplanted soil (Wei et al. 2019, Yang et al. 2023). Finally, ecological theory and experiments suggest that biodiversity correlates positively with ecosystem functioning and this pattern has been also shown to hold in terrestrial ecosystems (Pennekamp et al. 2018, Jochum et al. 2020). Phage diversity could, hence be important by promoting bacterial community stability and providing a more diverse suite of AMGs and accessory genome functions for the bacterial and plant community. To address all these questions, more research on phage ecology and evolution in terrestrial ecosystems is required where phages are recognized as a vital component of soil microbiomes with clear links with human and plant compartments within the One Health framework.
